# Racial/ethnic and neighbourhood social vulnerability disparities in COVID-19 testing positivity, hospitalization, and in-hospital mortality in a large hospital system in Pennsylvania: A prospective study of electronic health records

**DOI:** 10.1016/j.lana.2022.100220

**Published:** 2022-03-03

**Authors:** Usama Bilal, John B. Jemmott, Alina Schnake-Mahl, Kathleen Murphy, Florence Momplaisir

**Affiliations:** a Department of Epidemiology and Biostatistics and Urban Health Collaborative, Drexel Dornsife School of Public Health, 3600 Market St, Room 730, Philadelphia, PA 19104, USA; b Urban Health Collaborative, Drexel Dornsife School of Public Health, Philadelphia, PA, USA; c Annenberg School for Communication, University of Pennsylvania, Philadelphia, PA, USA; d University of Pennsylvania Perelman School of Medicine, Department of Psychiatry, PA, USA; e University of Pennsylvania Perelman School of Medicine, Division of Infectious Diseases, PA, USA; f Leonard Davis Institute of Health Economics, University of Pennsylvania, Philadelphia, PA, USA

**Keywords:** COVID-19, Health disparities, Neighborhoods, Electronic health records

## Abstract

**Background:**

Disparities in COVID-19 mortality by race/ethnicity or neighborhood have been documented using surveillance data. We aimed to describe disparities by race/ethnicity and neighbourhood social vulnerability in COVID-19 positivity, hospitalization, and mortality.

**Methods:**

We obtained data from the electronic health records of all individuals who tested positive for COVID-19 in the University of Pennsylvania Health System (UPHS) or were hospitalized with confirmed COVID-19 infection in five UPHS hospitals from March 1, 2020, to March 31, 2021. The main predictors were race/ethnicity and neighbourhood-level social vulnerability. The main outcomes were COVID-19 test positivity, hospitalization with COVID-19, and 30-day in-hospital mortality following hospitalization with COVID-19.

**Findings:**

A total of 225,129 unique individuals received COVID-19 testing and 18,995 had a positive test result. A total of 5,794 unique patients were hospitalized with COVID-19 and 511 died in-hospital within 30 days. Racial/ethnic minority groups and residents of higher social vulnerability neighbourhoods had higher test positivity and risk of hospitalization. We did not see in-hospital mortality disparities during the first wave but observed 75% and 68% higher odds of death among Hispanic and Asians compared to Whites during subsequent waves.

**Interpretation:**

We observed significant racial/ethnic and neighbourhood disparities in COVID-19 outcomes, especially test positivity and odds of hospitalization, highlighting the importance of equitably improving access to preventive measures to reduce SARS-CoV-2 infection, including reducing exposure to the virus and ensuring equity in vaccination.

**Funding:**

National Institutes of Health.

**Research in context**

**Evidence before this study**

COVID-19 surveillance data has shown wide racial/ethnic and neighborhood disparities in COVID-19 outcomes, including incidence, hospitalization, and mortality. Reports using individual-level data from other health systems have shown similar disparities for incidence and hospitalization, especially by race/ethnicity, but much narrower or inexistent disparities in mortality once hospitalized.

**Added value of this study**

In this study of more than 200,000 tested individuals and almost 6,000 hospitalized COVID-19 patients in a large academic health system, racial/ethnic minorities and residents of high social-vulnerability neighborhoods had higher odds of COVID-19 test positivity and hospitalization. We did not observe disparities in in-hospital mortality by social vulnerability and saw few instances of increased mortality among specific racial/ethnic groups. We also found a compounding effect so that racial/ethnic minorities living in higher social vulnerability areas had a further increased risk of testing positive or being hospitalized after testing positive for COVID-19.

**Implications of all the available evidence**

Given the preponderance of disparities in a proxy for incidence (testing positivity) and in risk of hospitalization, and the much less clear disparities in in-hospital mortality, these results highlight the importance of reducing disparities in exposure to and transmission of SARS-CoV-2, including ensuring equity in vaccination.

Alt-text: Unlabelled box

## Introduction

The COVID-19 pandemic has disproportionately impacted racial/ethnic minorities and people living in low socioeconomic status areas, who have suffered higher rates of test positivity, infection, hospitalization, and mortality.^1^, ^2^, ^3^, ^4^, ^5^, ^6^ During 2020, there was excess all-cause mortality among non-Hispanic Black (NHB), Indigenous, and Latino individuals in the U.S., double that among non-Hispanic White (NHW) and non-Hispanic Asians (NHA) individuals,^3^ mostly due to deaths coded as COVID-19^3^. Inequities in COVID-19 outcomes emerge from disparities in exposure to SARS-CoV-2 and vulnerability to severe COVID-19 disease.^7^ However, the policy implications of reducing disparities in exposure and vulnerability may be different.

Multiple factors drive differential risk of exposure to COVID-19. Occupational exposures to an infected person in the workplace have resulted from a limited ability (or inability) to work remotely, inadequate access to personal protective equipment (PPE), and challenges with socially distancing in the workplace.^8^ Differential environmental exposures have been influenced by housing conditions, including overcrowding or multigenerational/multifamily households.^7^^,^^9^ These drivers of differential exposure put racial/ethnic minorities at a disadvantage due to the patterns generated by the system of racial capitalism^10^^,^^11^ and racial residential segregation,^12^ the product of centuries of structural racism.^13^ Disparities in vulnerability to severe COVID-19 disease reflect differences in health care access and quality and the prevalence of risk factors for severe COVID-19, including respiratory and metabolic diseases,^7^^,^^10^ which are also driven by systems of inequity and racism.^7^^,^^14^

Disparities in COVID-19 mortality by race/ethnicity or socioeconomic status (SES) have been repeatedly documented at the population-level.^1^, ^2^, ^3^, ^4^ However, research documents that most of these disparities originate from a disparate risk of infection, with no disparities in age-adjusted in-hospital mortality once infected.^15^, ^16^, ^17^ Most studies of in-hospital mortality were conducted in unique integrated networks (Kaiser Permanente and the Veterans Affairs Administration), so the results may not be generalizable to other settings.^15^^,^^17^ Moreover, to our knowledge, studies exploring racial/ethnic inequities in COVID-19 outcomes have not simultaneously explored potential interactions with neighbourhood-level social vulnerability.^1^, ^2^, ^3^, ^4^^,^^15^^,^^17^ The objective of this study is to describe disparities by race/ethnicity and neighbourhood social vulnerability in COVID-19 test positivity, hospitalization, and in-hospital mortality, leveraging individual-level data from over 200,000 tested individuals and almost 6000 hospitalized individuals with COVID-19 within a large Pennsylvania health system. We hypothesized that racial/ethnic minorities and individuals living in neighbourhoods with higher social vulnerability would be at increased risk of COVID-19 test positivity, hospitalization, and in-hospital mortality.

## Methods

### Study setting

We used data from the University of Pennsylvania Health System (UPHS), a system of six hospitals and several specialty centres and outpatient clinics, all in Philadelphia or surrounding counties in Pennsylvania and New Jersey, that serves patients regardless of ability to pay. The Penn Data Warehouse houses a repository of integrated data from electronic health records (EHR) provided by many sources (including inpatient and outpatient orders, test results, and documentation).

We included data from two separate cohorts (*Appendix Fig.* 1 details the sample sizes for each analysis). The testing cohort includes all individuals tested for COVID-19 in the UPHS from March 1st 2020 to March 31st 2021. While patients were required to have an order from a UPHS physician to receive a COVID-19 test, a few testing sites allowed walk-up testing for the community. Variables include test collection date, age, sex, race-ethnicity, and neighbourhood of residence (zip code).

The hospitalization cohort includes all patients hospitalized in the UPHS system from March 1st, 2020 to March 31st, 2021 with a “COVID-19 Confirmed” flag. The “COVID-19 Confirmed” infection flag was reviewed and ordered in the EHR by providers and Infection Control team members for infection control purposes to reflect hospitalized patients with confirmed acute COVID-19 infection. This flag was implemented on March 20th, 2020 and applied retroactively to hospitalizations that had not been discharged by that date. Importantly, this flag is not exclusive of individuals hospitalized because of COVID-19; it reflects hospitalized individuals who have acute COVID-19 regardless of the reason for their hospitalization. Variables for this cohort, beyond the ones for testing above, include dates of admission and discharge, vital status at discharge (dead or alive, regardless of whether patients were discharged to home or hospice care), and intensive care unit (ICU) stay or use of invasive mechanical ventilation.

### Outcomes

We defined three primary outcomes, all defined at the individual level. First, test positivity from the testing cohort; for this, we created a dummy variable indicating whether each unique individual ever had a positive test, instead of including multiple positive test results. Second, risk of hospitalization among patients that tested positive for COVID-19 in the testing cohort; for this, we considered the first positive test, and explored whether patients were hospitalized 2 weeks before or 4 weeks after this test. The retrospective follow-up (2 weeks) allows for individuals who were hospitalized with an initial negative test on admission but later found to have COVID-19 during their hospitalisation and is consistent with the definition of hospitalizations with COVID-19 by the New York City Department of Health, allowing for a maximal duration of community exposure and subsequent development of COVID-19 infection. The prospective follow-up (4 weeks) reflects a lag time between symptom onset and hospitalization.^18^ Third, we used 30-day in-hospital all-cause mortality^15^ as the main outcome for the hospitalisation cohort and explored two secondary outcomes as markers of in-hospital severity: ICU admission and invasive mechanical ventilation. These outcomes were considered only during the first hospitalization.

### Predictors

We used two key predictors: individual-level race/ethnicity and neighbourhood-level social vulnerability. Race/ethnicity was self-reported in the EHR as (1) NHB, (2) NHW, (3) Hispanics, (4) NHA, (5) all other race/ethnicity (NHO, including Native Americans, Pacific Islanders, multiracial, all pooled due to limited sample size), and (6) missing or declined. In our analysis, individual race/ethnicity is not a risk factor, but a proxy for the lived experiences of individuals under a societal racial stratification system, generating different outcomes for individuals in different racial/ethnic groups due to differential access to resources, segregation into neighbourhoods, discrimination, and other effects of structural racism.^3^^,^^7^^,^^10^, ^11^, ^12^, ^13^, ^14^

Neighbourhood social vulnerability was measured using the Social Vulnerability Index (SVI),^19^ created by the Centers for Disease Control and Prevention (CDC) to measure area-level vulnerability to natural disasters and pandemics by combining 15 census indicators in four domains: SES, household composition & disability, minority status & language, and housing type & transportation. We categorized patients into tertiles of SVI, using the entire sample of zip codes in our study. *Appendix* 1 contains a list of all variables included in the SVI along with cut-offs for the tertiles.

### Covariates

Covariates included patients age (in years), sex (male/female), and hospital of admission.

### Statistical analysis

We described the study sample characteristics by wave (1st wave, from pandemic onset to June 30th, 2020, vs subsequent waves). We then fitted multilevel logistic regression models of individuals nested in neighbourhoods to assess disparities in testing positivity, hospitalization, and in-hospital mortality, along with secondary outcomes of severity. For all models, we examined disparities by race/ethnicity and area-level social vulnerability in separate models, using NHW and low SVI as reference groups, and then combined them into a single model with interaction terms between race/ethnicity and SVI tertiles. All models were adjusted for age, age squared, sex, and hospital (for in-hospital outcomes). We also adjusted by wave and added interaction terms between wave and race/ethnicity and/or social vulnerability, to control for potential differences in patient management and thresholds for hospitalization over time.^20^^,^^21^ The joint statistical significance of interaction terms was assessed using the F test and comparing nested models with and without interaction terms (see *Appendix* 2 for details).

We conducted two sensitivity analyses for the in-hospital outcomes: (a) restricting these analyses to individuals testing positive for COVID-19 and subsequently hospitalized in the UPHS system (instead of all individuals hospitalized at the UPHS system with a COVID-19 confirmed flag, regardless of where they were tested); and (b) restricting these analyses to individuals admitted on March 20th, 2020, or later, when the “COVID-19 confirmed” flag was implemented. All analyses were conducted in R v4.1. The University of Pennsylvania Institutional Review Board approved these analyses with protocol #843605.

### Role of the funding source

The funding agency had no involvement in the study design; in the data collection, analyses or interpretation of data; in the writing of this work; or in the decision to submit the manuscript for publication.

## Results

Table 1 shows descriptive statistics for the sample. The final analytic samples (*Appendix Fig.* 1) included a total of 348,148 COVID-19 tests in 225,129 unique individuals (median [range] number of tests per individual = 1 [1-44]), living in 3,276 neighbourhoods. Around 59% of tested patients were females, 62% were <60 years of age, 58% were NHW, 25% were NHB, 5% were Hispanics, 5% were NHA, and 3% were NHO. Both test positivity and risk of hospitalization were higher during the first wave than subsequent waves. There were 5,794 unique hospitalized patients with a COVID-19 flag living in 528 neighbourhoods. About 52% were female, 44% were aged <60, 38% were NHW, 43% were NHB, 9% were Hispanic, 5% were NHA, and 3% were NHO. In-hospital mortality and likelihood of ICU stay and mechanical ventilation were higher during the first wave. We observed a sharp increase in the share of hospitalized patients that were NHW in subsequent waves (43%) compared with the first wave (30%) and a concurrent decrease in the share of NHB (49% vs 39%) and Hispanics (11% vs 8%).

**Table 1:** Characteristics of testing and hospitalization participants, by wave.

	Testing Cohort				Hospitalization Cohort			
	Overall	1^st^ wave	Subsequent waves	p-val*	Overall	1^st^ wave	Subsequent waves	p-val*
Unique Individuals	225129	60766	183823		5794	2045	3749	
Unique Neighborhoods	3276	1699	2924		528	283	454	
Positive test	7.3%	12.3%	5.9%	<0.001				
Hospitalized	20.3%	21.5%	19.8%	0.002				
Deceased					8.8%	13.1%	6.5%	<0.001
ICU Stay					27.9%	31.0%	26.2%	<0.001
Mech. Ventilation					12.9%	17.8%	10.2%	<0.001
Age (Median [IQR])	52 [35-66]	51 [35-65]	53 [35-67]	<0.001	62 [46-75]	62 [46-76]	62 [47-75]	0.018
Age Categories				<0.001				0.666
0-39	32.0%	33.7%	31.4%		18.2%	18.3%	18.2%	
40-59	30.0%	30.4%	29.9%		25.7%	25.6%	25.7%	
60-79	31.4%	29.3%	32.2%		39.1%	38.3%	39.5%	
80+	6.5%	6.6%	6.5%		17.0%	17.8%	16.6%	
Sex				0.012				0.722
Female	58.6%	58.2%	58.8%		52.2%	52.6%	52.0%	
Male	41.4%	41.8%	41.2%		47.8%	47.4%	48.0%	
Race/ethnicity				<0.001				<0.001
Non-Hispanic White	58.1%	53.5%	59.9%		38.4%	29.8%	43.1%	
Non-Hispanic Black	24.8%	28.8%	23.3%		42.6%	49.0%	39.1%	
Hispanic	4.5%	5.3%	4.3%		9.1%	11.3%	7.9%	
Non-Hispanic Asian	4.9%	4.7%	4.9%		5.5%	5.1%	5.8%	
Non-Hispanic Other	2.9%	3.1%	2.8%		2.7%	2.7%	2.7%	
Missing	4.8%	4.7%	4.8%		1.7%	2.0%	1.5%	
Social Vulnerability				<0.001				<0.001
Low	32.7%	30.0%	33.7%		22.7%	20.6%	23.9%	
Medium	28.0%	26.9%	28.4%		24.7%	21.5%	26.4%	
High	39.3%	43.1%	37.9%		52.6%	57.9%	49.7%	

⁎ =p-value comparing first and second and subsequent waves, using the χ^2^ test for differences in proportions or Kruskal-Wallis test for differences in medians (for age).

Across the study period, racial/ethnic minorities had higher odds of testing positivity than NHW (see Table 2; *Appendix Table* 2.1 shows the interaction coefficients). For example, during the first wave, the aOR for testing positivity among NHB, Hispanics, NHA, and NHO were 2.80 (95% CI 2.62-2.99), 4.22 (95% CI 3.85-4.62), 1.61 (95% CI 1.42-1.82), and 2.40 (95% CI 2.11-2.74), respectively, compared to NHW. These associations persisted during subsequent waves, although closer to the null. Residents of medium or high social-vulnerability areas also had higher odds of test positivity: the odds of a positive test during the first wave in medium and high social-vulnerability areas were 1.19 (95% CI 1.03-1.37) and 2.39 (95% CI 2.09-2.74) times higher than in low social-vulnerability areas. These associations were closer to the null during the subsequent waves but remained significant for high vs low social vulnerability areas (aOR=1.67, 95% CI 1.47-1.89).

**Table 2:** Odds ratios (95% CI) of testing positivity, hospitalization, and in-hospital mortality associated with race/ethnicity and neighborhood-level social vulnerability considered separately, by wave.

		Testing Positivity (OR, 95% CI)		Hospitalization (OR, 95% CI)		In-hospital Mortality (OR, 95% CI)	
	Category	1^st^ Wave	Subsequent Waves	1^st^ Wave	Subsequent Waves	1^st^ Wave	Subsequent Waves
Race Ethnicity	NH White	1 (Ref.)	1 (Ref.)	1 (Ref.)	1 (Ref.)	1 (Ref.)	1 (Ref.)
	NH Black	2.80 (2.62-2.99)	1.57 (1.49-1.65)	1.74 (1.48-2.05)	2.08 (1.84-2.35)	0.88 (0.63-1.22)	1.10 (0.79-1.54)
	Hispanic	4.22 (3.85-4.62)	2.17 (2.01-2.35)	1.82 (1.46-2.26)	1.81 (1.51-2.16)	0.76 (0.42-1.38)	1.75 (1.01-3.02)
	NH Asian	1.61 (1.42-1.82)	1.44 (1.32-1.57)	1.68 (1.24-2.28)	1.53 (1.25-1.87)	1.08 (0.59-1.98)	1.68 (1.01-2.79)
	NH Other	2.40 (2.11-2.74)	1.73 (1.56-1.92)	0.73 (0.49-1.07)	1.09 (0.83-1.42)	1.21 (0.55-2.66)	1.52 (0.67-3.45)
	Missing	2.31 (2.06-2.60)	1.32 (1.20-1.46)	0.33 (0.22-0.51)	0.48 (0.34-0.69)	1.27 (0.49-3.26)	3.86 (1.82-8.17)
	p-val*	<0.001		0.108		0.186	
Social Vulnerability	Low	1 (Ref.)	1 (Ref.)	1 (Ref.)	1 (Ref.)	1 (Ref.)	1 (Ref.)
	Medium	1.19 (1.03-1.37)	1.08 (0.95-1.23)	0.91 (0.68-1.22)	1.23 (0.96-1.57)	0.99 (0.67-1.46)	1.08 (0.76-1.54)
	High	2.39 (2.09-2.74)	1.67 (1.47-1.89)	1.34 (1.02-1.74)	1.85 (1.46-2.34)	0.95 (0.65-1.39)	1.05 (0.73-1.51)
	p-val*	<0.001		0.005		0.903	

models control for age, age^2^, sex, and hospital (for in-hospital mortality). Race/ethnicity and SVI results come from different models. Testing positivity refers to the odds of ever testing positive; hospitalization refers to the odds of being hospitalized 2 weeks prior or 4 weeks after the first positive test; in-hospital mortality refers to the odds of dying in-hospital by 30 days after admission with a COVID-19 confirmed flag.

⁎ p-val refers to the p-value for interaction between wave and race/ethnicity or SVI.

NHB, Hispanics, and NHA had significantly higher odds of hospitalization with COVID-19 than NHW, during both the first and subsequent waves (Table 2). During the first wave, the aORs for hospitalization among NHB, Hispanics, and NHA were 1.74 (95% CI 1.48-2.05), 1.82 (95% CI 1.46-2.26), and 1.68 (95% CI 1.24-2.28), respectively, compared to NHW. These associations were similar in magnitude in subsequent waves. Individuals with missing race/ethnicity had lower odds of hospitalization than NHW. In addition, the odds of hospitalization were significantly higher in high social-vulnerability areas during the first (aOR=1.34, 95% CI 1.02-1.74) and subsequent waves (aOR=1.85, 95% CI 1.46-2.34), with stronger associations during the subsequent waves. We found no significant disparities in 30-day in-hospital mortality among any racial/ethnic group during the first wave, but significantly higher mortality among Hispanics (aOR=1.75, 95% CI 1.01-3.02) and NHA (aOR=1.68, 95% CI 1.01-2.79) than NHW during subsequent waves. We did not observe any disparities in 30-day in-hospital mortality by levels of neighbourhood social vulnerability.

Table 3 shows the result of the analysis including race/ethnicity, neighbourhood-level SVI, and their interaction in the same models (*Appendix Tables* 2.2, 2.3, and 2.4 show the interaction coefficients themselves). The interactions between race/ethnicity and SVI were significant for testing positivity and hospitalization. For example, during the first wave and compared to NHW living in low social-vulnerability areas, Hispanics and NHB living in high social-vulnerability areas had 6.5 times and 4 times the odds of testing positive and 1.9 times the odds of hospitalization. Moreover, NHW living in high social-vulnerability areas had significantly higher odds of testing positive and hospitalization than NHW living in low social-vulnerability areas. Also, within each tertile of neighbourhood-level social vulnerability, the odds of test positivity and hospitalization were higher among racial/ethnic minorities than NHW. For in-hospital mortality, we did not observe any major departures from previous models.

**Table 3:** Odds ratios (95% CI) of testing positivity, hospitalization, and in-hospital mortality associated with race/ethnicity and neighborhood-level social vulnerability considered simultaneous, by wave.

		1^st^ wave			Subsequent Waves		
	Race Ethnicity	Low SVI	Middle SVI	High SVI	Low SVI	Middle SVI	High SVI
**Testing Positivity**	NH White	1 (Ref.)	0.99 (0.85-1.14)	1.31 (1.12-1.53)	1 (Ref.)	1.04 (0.92-1.18)	1.43 (1.26-1.63)
	NH Black	2.68 (2.24-3.21)	2.75 (2.32-3.26)	3.96 (3.45-4.54)	1.63 (1.40-1.90)	1.67 (1.43-1.94)	2.29 (2.02-2.60)
	Hispanic	2.59 (2.00-3.35)	4.01 (3.24-4.97)	6.46 (5.50-7.58)	2.52 (2.11-3.02)	2.54 (2.11-3.06)	2.90 (2.50-3.37)
	NH Asian	1.00 (0.77-1.30)	1.54 (1.19-2.00)	2.87 (2.33-3.54)	0.86 (0.72-1.04)	1.36 (1.11-1.67)	2.91 (2.48-3.43)
	Other	1.89 (1.41-2.53)	2.56 (1.93-3.40)	3.50 (2.85-4.31)	1.62 (1.31-2.00)	1.60 (1.26-2.03)	2.72 (2.28-3.25)
	Missing	1.75 (1.35-2.26)	1.63 (1.25-2.12)	4.09 (3.39-4.93)	1.36 (1.13-1.64)	1.39 (1.14-1.69)	1.85 (1.54-2.23)
	p-val*	<0.001			<0.001		
	p-val⁎⁎	<0.001					
**Hospitalization**	NH White	1 (Ref.)	0.71 (0.51-1)	1.17 (0.83-1.64)	1 (Ref.)	1.21 (0.94-1.55)	1.41 (1.09-1.82)
	NH Black	1.41 (0.91-2.18)	1.59 (1.10-2.31)	1.87 (1.41-2.48)	1.97 (1.42-2.74)	2.52 (1.86-3.42)	2.75 (2.16-3.50)
	Hispanic	1.57 (0.84-2.91)	1.97 (1.26-3.10)	1.85 (1.32-2.59)	1.85 (1.23-2.80)	1.62 (1.08-2.43)	2.67 (1.98-3.60)
	NH Asian	2.39 (1.30-4.40)	1.58 (0.86-2.90)	1.46 (0.90-2.35)	1.85 (1.23-2.80)	1.80 (1.16-2.79)	1.90 (1.35-2.67)
	Other	1.00 (0.46-2.17)	1.10 (0.55-2.19)	0.45 (0.23-0.87)	1.14 (0.65-1.98)	1.42 (0.80-2.51)	1.40 (0.92-2.11)
	Missing	0.28 (0.11-0.71)	0.13 (0.05-0.33)	0.50 (0.29-0.87)	0.33 (0.15-0.75)	0.29 (0.13-0.64)	1.08 (0.67-1.76)
	p-val*	0.011			0.019		
	p-val⁎⁎	0.268					
**In-hospital mortality**	NH White	1 (Ref.)	1.21 (0.75-1.96)	0.96 (0.52-1.80)	1 (Ref.)	0.99 (0.64-1.53)	0.97 (0.56-1.67)
	NH Black	1.57 (0.73-3.37)	0.72 (0.35-1.49)	0.91 (0.57-1.43)	1.22 (0.49-2.99)	1.15 (0.59-2.24)	1.06 (0.67-1.68)
	Hispanic	0.55 (0.07-4.50)	0.55 (0.19-1.64)	1.05 (0.49-2.24)	2.98 (1.07-8.27)	2.02 (0.81-5.01)	1.18 (0.48-2.88)
	NH Asian	0.65 (0.21-1.97)	1.42 (0.52-3.85)	1.65 (0.61-4.51)	1.07 (0.40-2.88)	2.01 (0.87-4.61)	1.94 (0.85-4.42)
	Other	1.41 (0.41-4.85)	1.17 (0.31-4.45)	1.22 (0.25-5.88)	0.62 (0.08-4.86)	4.14 (1.10-15.56)	1.26 (0.36-4.35)
	Missing	0.83 (0.09-7.70)	1.11 (0.12-10.03)	1.65 (0.50-5.45)	3.71 (0.91-15.13)	2.16 (0.45-10.33)	5.33 (1.83-15.51)
	p-val*	0.641			0.818		
	p-val⁎⁎	0.783					

models control for age, age^2^, sex, and hospital (for in-hospital mortality) and including an interaction between race/ethnicity and SVI. Testing positivity refers to the odds of ever testing positive; hospitalization refers to the odds of being hospitalized 2 weeks prior or 4 weeks after the first positive test; in-hospital mortality refers to the odds of dying in-hospital by 30 days after admission with a COVID-19 confirmed flag.

⁎ p-val refers to the global test for interaction between the race/ethnicity and SVI coefficients;

⁎⁎ p-val refers to the global test for the three way interactions between race/ethnicity, SVI, and wave.

**Figure 1:**
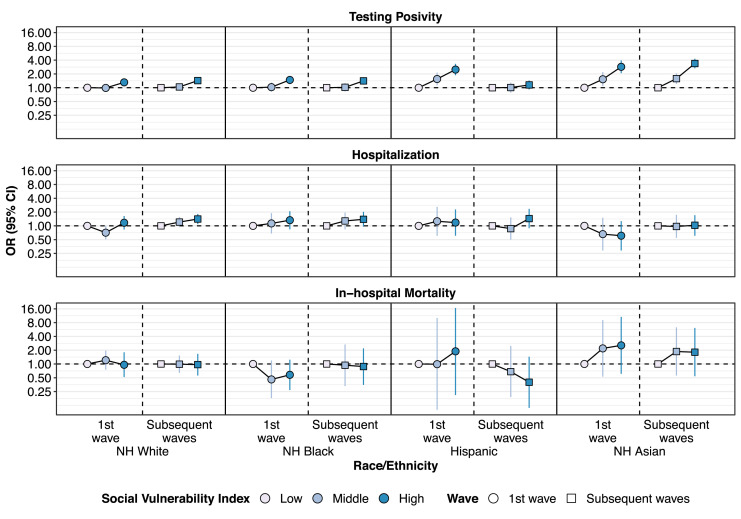
Odds ratios (95% CI) of testing positivity, hospitalization, and in-hospital mortality associated with neighbourhood-level social vulnerability across racial/ethnic groups, by wave. models control for age, age^2^, sex, and hospital (for in-hospital mortality) and include an interaction between race/ethnicity and SVI. Testing positivity refers to the odds of ever testing positive; hospitalization refers to the odds of being hospitalized 2 weeks prior or 4 weeks after the first positive test; in-hospital mortality refers to the odds of dying in-hospital by 30 days after admission with a COVID-19 confirmed flag. To facilitate comparisons, we exclude NHO and people with missing race/ethnicity information from this figure given wide confidence intervals. The figure includes 95% confidence intervals for ORs, but some may not be visible if they are too narrow (e.g., for NH Whites and testing).

Figure 1 shows results from the same model but allows for comparisons between strata of social vulnerability by race/ethnicity (*Appendix Table* 2.5 shows the results of testing the null hypothesis of no differences between waves in the association between social vulnerability and the outcomes by race/ethnicity). We found that Hispanics had a stronger association between social vulnerability and test positivity in the first wave than subsequent waves, while this association did not vary drastically by wave for the other racial/ethnic groups. We also found that associations between social vulnerability and risk of hospitalization varied between the first and subsequent waves for all racial/ethnic groups. For example, we found no clear association between risk of hospitalization and SVI during the first wave for NHW, but a clear dose-response association during subsequent waves, while for NHA, there was an inverted association with SVI during the first wave and no association during subsequent waves. Last, we found no differences in the association between the SVI and in-hospital mortality between the first and subsequent waves for any racial/ethnic group.

Compared to NHW and people living in low social vulnerability areas, all racial/ethnic minority groups and people living in medium and high social vulnerability areas had higher odds of ICU stay and mechanical ventilation during the first wave, indicating heightened severity (Table 4). These associations generally weakened during subsequent waves, especially for ICU stay. Last, *Appendix Tables* 3.1 and 3.2 present the sensitivity analysis results restricting the in-hospital outcomes analyses to individuals tested in the UPHS system and to hospitalizations from March 20th, 2020, onwards. The associations were consistent in direction and magnitude compared to the main analyses.

**Table 4:** Odds ratios (95% CI) of intensive care unit stay, and invasive mechanical ventilation associated with race/ethnicity and neighborhood-level social vulnerability, by wave.

		ICU Stay (OR, 95% CI)		Mech. Ventilation (OR, 95% CI)	
	Category	1^st^ Wave	Subsequent Waves	1^st^ Wave	Subsequent Waves
**Race Ethnicity**	NH White	1 (Ref.)	1 (Ref.)	1 (Ref.)	1 (Ref.)
	NH Black	1.4 (1.10-1.78)	0.94 (0.78-1.12)	1.20 (0.88-1.62)	0.89 (0.68-1.15)
	Hispanic	1.62 (1.13-2.30)	1.53 (1.15-2.04)	1.69 (1.10-2.60)	1.85 (1.24-2.74)
	NH Asian	1.76 (1.11-2.78)	0.87 (0.61-1.25)	2.14 (1.29-3.54)	1.24 (0.78-1.95)
	NH Other	1.58 (0.87-2.88)	0.89 (0.55-1.46)	1.54 (0.77-3.09)	1.46 (0.80-2.66)
	Missing	2.35 (1.21-4.56)	3.34 (1.93-5.77)	2.23 (1.08-4.61)	4.30 (2.38-7.76)
	p-val*	0.014		0.138	
**Social Vulnerability**	Low	1 (Ref.)	1 (Ref.)	1 (Ref.)	1 (Ref.)
	Medium	1.51 (1.10-2.07)	1.04 (0.84-1.28)	1.47 (1.00-2.16)	0.98 (0.73-1.33)
	High	1.64 (1.23-2.18)	0.86 (0.70-1.06)	1.17 (0.82-1.68)	0.76 (0.57-1.02)
	p-val*	<0.001		0.119	

models control for age, age^2^, sex, and hospital. Race/ethnicity and SVI results come from different models. Outcomes refer to the odds of ICU stay or invasive mechanical ventilation during the first hospitalization with a COVID-19 confirmed flag.

⁎ p-val refers to the p-value for interaction between wave and race/ethnicity or SVI.

## Discussion

This study analysing disparities in COVID-19 test positivity, hospitalization, in-hospital mortality, and COVID-19 severity indicators in a large health system in Pennsylvania resulted in four key findings. First, we found substantial racial/ethnic and neighbourhood social vulnerability disparities in testing positivity and risk of hospitalization with COVID-19. Second, these disparities were compounded when exploring racial/ethnic and neighbourhood disparities simultaneously, as NHB and Hispanics in high social-vulnerability areas had 4–7 times higher odds of testing positivity and 2.7 times higher odds of hospitalization than NHW in low social-vulnerability areas. Third, in-hospital mortality after COVID-19 hospitalization decreased across waves, had no clear disparities during the first wave and was highest among Hispanics and NHA during the subsequent waves. Fourth, we found higher severity (as measured by ICU stay and mechanical ventilation) for most racial/ethnic minorities and individuals living in higher social-vulnerability areas, especially during the first wave, compared to NHW and individuals living in low social vulnerability areas. In summary, racial/ethnic minorities and individuals living in higher social vulnerability areas had higher odds of testing positive for or being hospitalized with COVID-19 and higher severity once hospitalized, while the odds of mortality once hospitalized varied by specific racial/ethnic group.

Our results concerning test positivity and risk of hospitalization mirror previous studies using individual- and aggregate-level data. In several studies, NHB and Hispanics showed higher test positivity^15^, ^16^, ^17^ and hospitalization^22^ than NHW, while areas of higher social vulnerability, segregation, or socioeconomic deprivation also had higher test positivity.^2^ Two of the main drivers of SARS-CoV-2 transmission disparities are occupational and household exposures^7^ originating from a system of racial capitalism^10^^,^^11^ that increases the likelihood that racial/ethnic minorities and low-SES individuals work in occupations deemed essential with limited access to telework^9^ and live in overcrowded and multigenerational households.^9^ Possible reasons behind the higher risk of hospitalization include elevated vulnerability to severe COVID-19 infection owing to a higher prevalence of known comorbid risk factors for more severe disease, including diabetes and chronic cardiovascular and respiratory conditions.^23^ We did not adjust for comorbidities as these may be part of the pathway between racism or social vulnerability and COVID-19 outcomes.

We did not find a clear pattern regarding in-hospital mortality. During the first wave, the odds of in-hospital mortality were similar across racial/ethnic groups and levels of neighbourhood social vulnerability, while during subsequent waves, the odds were higher among Hispanics and NHA. The presence of higher mortality among NHA and the lack of higher mortality among NHB conflict with other reports examining COVID-19 mortality using surveillance data^3^^,^^5^^,^^22^^,^^24^ but are consistent with other studies showing a lack of disparities (or even inverted disparities) in disease severity and in-hospital mortality.^15^^,^^17^^,^^25^ This conflicting pattern may be due to collider stratification bias,^26^ a form of selection bias occurring when a selection factor (in this case, hospitalization) is affected by both the predictor of interest (here, race/ethnicity or neighbourhood social vulnerability) and another unmeasured cause of the outcome.^26^ Consequently, the characteristics of racial/ethnic minorities and people living in high social-vulnerability areas who end up hospitalized would be different than those of NHW or people living in low social-vulnerability areas. Adapting an example from Griffith et al.^26^: (a) if high social vulnerability causes a higher risk of hospitalization; (b) if individuals with more access to healthcare are more likely to be hospitalized (holding severity constant) while having a lower mortality risk; then (c) hospitalized individuals will be systematically different from non-hospitalized individuals, weakening the apparent SVI-to-mortality association. As area-(or individual-)level social vulnerability potentially drives access to healthcare, adjusting for access to healthcare would also fail to produce unbiased estimates of this association. There may also be a competing risk (discharge), where racial/ethnic minorities and people living in higher social vulnerability areas are more likely to die at home.^25^ Since we could not ascertain vital status after discharge, this remains a possibility. The second set of explanations may be an actual lack of disparities once hospitalized. Regular review and implementation of evidence-based COVID-19 treatment protocols for hospitalized patients^21^ (including use of steroids and other immunomodulatory agents depending on illness severity and increased use of non-invasive ventilation strategies and patient proning) may have contributed to health equity by standardizing care of all patients admitted with COVID-19. The UPHS system previously reported a halving of mortality risk among hospitalized COVID-19 patients during the first two months of the pandemic.^21^

We acknowledge some limitations. First, we were limited to examining in-hospital mortality, as vital status ascertainment outside of hospitalization is incomplete. If race/ethnicity or neighbourhood social vulnerability affects the place of death for hospitalized patients (e.g., different likelihood of being discharged), we may obtain biased associations due to competing risks (i.e., discharge acting as a competing risk for in-hospital mortality).^25^ Moreover, we could not ascertain vaccination status or use of newly authorized treatments (e.g., monoclonal antibodies), which may have contributed to some of the inequalities observed in the latter part of our study. However, given the existing disparities in both the administration of monoclonal antibodies^27^ and for vaccination,^28^ receipt of these therapies is likely on the causal pathway between racism or social vulnerability and COVID-19 outcomes. In the case of vaccines, by March 31st 2021 only 18% of the population of Philadelphia had been fully vaccinated.^29^ Similarly, we had no information on race/ethnicity for 4.8% of tested individuals and 1.7% of hospitalized individuals but decided to report positivity, hospitalization, and in-hospital mortality estimates for this group. Second, not all patients tested in this health system and requiring a subsequent hospitalization were hospitalized within the University of Pennsylvania Health System, leading to bias if the place of hospitalization differs by race/ethnicity or social vulnerability. Our sensitivity analysis restricting the in-hospital mortality analysis to individuals tested at the UPHS system (as a proxy for receiving care in the system) showed stronger associations during the subsequent waves, indicating that this may be a potential source of bias. Third, access to testing was especially limited during the first wave, potentially biasing positivity results towards groups with a higher proportion of symptomatic individuals (main type of tested patients during the first wave) or lower access to testing (lower number of tested individuals). Last, our results do not apply to the summer 2021 Delta variant wave, which became dominant in Philadelphia around July 2021, or the fall 2021 Omicron variant wave, which became dominant in December 2021.^30^

## Conclusion

In this large sample covering the population of a large hospital system in Pennsylvania, we demonstrated racial/ethnic and neighbourhood socioeconomic disparities in COVID-19 test positivity and hospitalization, while results for in-hospital mortality varied by race/ethnicity. The vast disparities we observed in test positivity and hospitalization point at the importance of reducing the risk of exposure to SARS-CoV-2 across both racial/ethnic minorities and people living in high social vulnerability areas to reduce inequities in COVID-19 outcomes.

## Contributions

UB, JBJ, and FM conceived the study. UB conducted the statistical analysis and wrote the first version of the draft with support from JBJ and FM. KM and ASM contributed to the interpretation of findings. All authors reviewed and approved the last version of the manuscript.

## Data availability statement

Data can be requested from the Penn Data Warehouse.

## Declaration of interests

Alina Schnake-Mahl holds equity in and consults for Cityblock Health. The other authors declare no conflict of interest

## Appendix Supplementary materials

Image, application 1

Image, application 2
