# Supplementary material for: Racial/ethnic and neighbourhood social vulnerability disparities in COVID-19 testing positivity, hospitalization, and in-hospital mortality in a large hospital system in Pennsylvania: A prospective study of electronic health records
Source: Lancet Reg Health Am. 2022 Mar 3;10:100220. doi: 10.1016/j.lana.2022.100220 (PMC8891851; doi:10.1016/j.lana.2022.100220)
Supplement: Supplementary file 1 [file mmc1.docx]

**Appendix 1: social vulnerability index and its components**

**Appendix 2: interaction analysis**

**Appendix 3: results of sensitivity analyses**
