# Supplementary material for: Racial/ethnic and neighbourhood social vulnerability disparities in COVID-19 testing positivity, hospitalization, and in-hospital mortality in a large hospital system in Pennsylvania: A prospective study of electronic health records
Source: Lancet Reg Health Am. 2022 Mar 3;10:100220. doi: 10.1016/j.lana.2022.100220 (PMC8891851; doi:10.1016/j.lana.2022.100220)
Supplement: Supplementary file 2 [file mmc2.docx]

**Appendix 1: social vulnerability index and its components**

The social vulnerability index is a measure of social vulnerability, defined by the CDC as “The degree to which a community exhibits certain social conditions, including high poverty, low percentage of vehicle access, or crowded households, may affect that community’s ability to prevent human suffering and financial loss in the event of disaster.” The index was created by the CDC by creating percentiles of the distribution of 15 variables in four domains, and then summing the percentiles. The overall ranking was created by adding up the four domains. A higher value of the index reflects higher social vulnerability. All data was obtained from the 2014-2018 5-year American Community Survey (ACS).

**Appendix Table 1.1: Components of the Social Vulnerability Index by Domain**

| **Domain** | **Variable** |
| --- | --- |
| **Socioeconomic Status** | Below Poverty |
|  | Unemployed |
|  | Income |
|  | No High School Diploma |
| **Household Composition & Disability** | Aged 65 or Older |
|  | Aged 17 or Younger |
|  | Civilian with a Disability |
|  | Single-Parent Households |
| **Minority Status & Language** | Minority |
|  | Speaks English “Less than Well” |
| **Housing Type & Transportation** | Multi-Unit Structures |
|  | Mobile Homes |
|  | Crowding |
|  | No Vehicle |
|  | Group Quarters |

We created tertiles of the SVI for the testing and hospitalization cohorts separately, to reflect the different number of included neighborhoods in each.

**Appendix Table 1.2: Cut-points and number of individuals by tertile.**

| **Tertile** | **Testing Cohort** | **Hospitalization Cohort** |
| --- | --- | --- |
| **Low** | 0.004-0.255;n=73600 | 0.010-0.249;n=1318 |
| **Middle** | 0.255-0.478;n=63068 | 0.249-0.501;n=1429 |
| **High** | 0.478-0.991;n=88461 | 0.502-0.989;n=3047 |

Footnote: Values are range (min-max) and number of individuals in each tertile. Number of individuals differs by tertile, as tertiles were created by neighborhood, not by individual.

**Appendix 2: interaction analysis**

To examine whether there were statistically significant interactions, we employed the following procedure. Note that we have simplified the model here to make the explanation more straightforward.

- The actual models use include adjustment for age, age^2^, sex, and hospital (for in-hospital mortality), along with a random intercept for ZCTA.
- Moreover, here we have only included two comparison categories for race/ethnicity (Black and Hispanic vs white)

For analysis in Table 2 (interaction between race/ethnicity and wave, or between SVI and wave)

- Full model: $logit\left( P Positive Test=1 \right)=\beta_{0}+\beta_{1}*SVI_{m}+\beta_{2}SVI_{h}+\beta_{3}Wave_{2}+\beta_{4}SVI_{m}*Wave_{2}+\beta_{5}\mathrm{SV}I_{h}*Wave_{2}$
- Nested model: $logit\left( P Positive Test=1 \right)=\beta_{0}+\beta_{1}*SVI_{m}+\beta_{2}SVI_{h}+\beta_{3}Wave_{2}$
- Therefore, a nested-model comparison of both models tests the null hypothesis that B4 and B5 are = 0 (no interaction by wave). The same type of model was fitted for race/ethnicity. The p-values from these joint F-tests are shown in Table 2.

For analysis in Table 3 (interaction between race/ethnicity, SVI, and wave)

- Full Model: $logit\left( P Positive Test=1 \right)=\beta_{0}+\beta_{1}*SVI_{m}+\beta_{2}SVI_{h}+\beta_{3}*Black+\beta_{4}Hispanic+\beta_{5}Wave_{2}+\beta_{6}SVI_{m}*Wave_{2}+\beta_{7}\mathrm{SV}I_{h}*Wave_{2}+\beta_{8}Black*Wave_{2}+\beta_{9}Hispanic*Wave_{2}+\beta_{10}SVI_{m}*Black+\beta_{11}\mathrm{SV}I_{h}*Black+\beta_{12}SVI_{m}*Hispanic+\beta_{13}\mathrm{SV}I_{h}*Hispanic+\beta_{14}SVI_{m}*Black*Wave_{2}+\beta_{15}SVI_{h}*Black*Wave_{2}+\beta_{16}SVI_{m}*Hispanic*Wave_{2}+\beta_{17}SVI_{h}*Hispanic*Wave_{2}$
- Nested model 1: $logit\left( P Positive Test=1 \right)=\beta_{0}+\beta_{1}*SVI_{m}+\beta_{2}SVI_{h}+\beta_{3}*Black+\beta_{4}Hispanic+\beta_{5}Wave_{2}+\beta_{6}SVI_{m}*Wave_{2}+\beta_{7}\mathrm{SV}I_{h}*Wave_{2}+\beta_{8}Black*Wave_{2}+\beta_{9}Hispanic*Wave_{2}+\beta_{10}SVI_{m}*Black+\beta_{11}\mathrm{SV}I_{h}*Black+\beta_{12}SVI_{m}*Hispanic+\beta_{13}\mathrm{SV}I_{h}*Hispanic+\beta_{14}SVI_{m}*Black*Wave_{2}+\beta_{15}SVI_{h}*Black*Wave_{2}+\beta_{16}SVI_{m}*Hispanic*Wave_{2}+\beta_{17}SVI_{h}*Hispanic*Wave_{2}$
  - This model excludes the three way interaction (race x SVI x wave)
- Nested model 2: $logit\left( P Positive Test=1 \right)=\beta_{0}+\beta_{1}*SVI_{m}+\beta_{2}SVI_{h}+\beta_{3}*Black+\beta_{4}Hispanic+\beta_{5}Wave_{2}+\beta_{6}SVI_{m}*Wave_{2}+\beta_{7}\mathrm{SV}I_{h}*Wave_{2}+\beta_{8}Black*Wave_{2}+\beta_{9}Hispanic*Wave_{2}+\beta_{10}SVI_{m}*Black+\beta_{11}\mathrm{SV}I_{h}*Black+\beta_{12}SVI_{m}*Hispanic+\beta_{13}\mathrm{SV}I_{h}*Hispanic+\beta_{14}SVI_{m}*Black*Wave_{2}+\beta_{15}SVI_{h}*Black*Wave_{2}+\beta_{16}SVI_{m}*Hispanic*Wave_{2}+\beta_{17}SVI_{h}*Hispanic*Wave_{2}$
  - This model excludes the three way interaction (race x SVI x wave) and the svi x race interaction
- Nested model 3: $logit\left( P Positive Test=1 \right)=\beta_{0}+\beta_{1}*SVI_{m}+\beta_{2}SVI_{h}+\beta_{3}*Black+\beta_{4}Hispanic+\beta_{5}Wave_{2}+\beta_{6}SVI_{m}*Wave_{2}+\beta_{7}\mathrm{SV}I_{h}*Wave_{2}+\beta_{8}Black*Wave_{2}+\beta_{9}Hispanic*Wave_{2}+\beta_{10}SVI_{m}*Black+\beta_{11}\mathrm{SV}I_{h}*Black+\beta_{12}SVI_{m}*Hispanic+\beta_{13}\mathrm{SV}I_{h}*Hispanic+\beta_{14}SVI_{m}*Black*Wave_{2}+\beta_{15}SVI_{h}*Black*Wave_{2}+\beta_{16}SVI_{m}*Hispanic*Wave_{2}+\beta_{17}SVI_{h}*Hispanic*Wave_{2}$
  - This model excludes interactions with wave (both svi x wave and race x wave)
- Nested model 4: $logit\left( P Positive Test=1 \right)=\beta_{0}+\beta_{1}*SVI_{m}+\beta_{2}SVI_{h}+\beta_{3}*Black+\beta_{4}Hispanic+\beta_{5}Wave_{2}+\beta_{6}SVI_{m}*Wave_{2}+\beta_{7}\mathrm{SV}I_{h}*Wave_{2}+\beta_{8}Black*Wave_{2}+\beta_{9}Hispanic*Wave_{2}+\beta_{10}SVI_{m}*Black+\beta_{11}\mathrm{SV}I_{h}*Black+\beta_{12}SVI_{m}*Hispanic+\beta_{13}\mathrm{SV}I_{h}*Hispanic+\beta_{14}SVI_{m}*Black*Wave_{2}+\beta_{15}SVI_{h}*Black*Wave_{2}+\beta_{16}SVI_{m}*Hispanic*Wave_{2}+\beta_{17}SVI_{h}*Hispanic*Wave_{2}$
  - This model excludes all interactions
- Therefore, the following comparisons of nested models result in the following hypothesis being tested:
  - **Nested 1 vs Nested 2, or the joint-test that B10-B13 are = 0: lack of SVI x race interaction within the first wave**
  - **Full vs Nested 2, or the joint-test that B10-B17 are = 0: lack of SVI x race interaction within the subsequent wave**
  - **Full vs Nested 1, or the joint-test that B14-B17 = 0: SVI x race interactions are similar between waves**
- Furthermore, we also explored, in the context of Figure 1, whether within each race/ethnic group, SVI-based disparities varied by wave. This was done by testing the joint hypothesis of B6 and B7 = 0 (for NHW), or B6, B7, B14, and B15 = 0 (for NHB), etc.

**Appendix Table 2.1: Interaction coefficients (Ratio of odds ratios) (95% CI) for testing positivity, hospitalization, and in-hospital mortality, between wave and race/ethnicity or social vulnerability.**

| **Outcome** | **Coef*** | **Race/Ethnicity** | **SVI** | **Wave** | **ROR (95% CI)** |
| --- | --- | --- | --- | --- | --- |
| **Testing Positivity** | B4 | . | Middle | Subsequent | 0.91 (0.83-1) |
|  | B5 | . | High | Subsequent | 0.7 (0.64-0.76) |
|  | ~B4/B5 | NHB | . | Subsequent | 0.56 (0.52-0.6) |
|  | ~B4/B5 | Hispanic | . | Subsequent | 0.51 (0.46-0.58) |
|  | ~B4/B5 | NHA | . | Subsequent | 0.9 (0.77-1.04) |
|  | ~B4/B5 | NHO | . | Subsequent | 0.72 (0.61-0.85) |
|  | ~B4/B5 | Missing | . | Subsequent | 0.57 (0.49-0.66) |
| **Hospitalization** | B4 | . | Middle | Subsequent | 1.35 (1.06-1.72) |
|  | B5 | . | High | Subsequent | 1.39 (1.13-1.69) |
|  | ~B4/B5 | NHB | . | Subsequent | 1.19 (1-1.42) |
|  | ~B4/B5 | Hispanic | . | Subsequent | 0.99 (0.76-1.3) |
|  | ~B4/B5 | NHA | . | Subsequent | 0.91 (0.63-1.3) |
|  | ~B4/B5 | NHO | . | Subsequent | 1.5 (0.94-2.39) |
|  | ~B4/B5 | Missing | . | Subsequent | 1.46 (0.85-2.52) |
| **In-hospital mortality** | B4 | . | Middle | Subsequent | 1.09 (0.65-1.82) |
|  | B5 | . | High | Subsequent | 1.11 (0.7-1.75) |
|  | ~B4/B5 | NHB | . | Subsequent | 1.26 (0.82-1.94) |
|  | ~B4/B5 | Hispanic | . | Subsequent | 2.3 (1.03-5.09) |
|  | ~B4/B5 | NHA | . | Subsequent | 1.55 (0.71-3.37) |
|  | ~B4/B5 | NHO | . | Subsequent | 1.26 (0.41-3.91) |
|  | ~B4/B5 | Missing | . | Subsequent | 3.05 (0.92-10.12) |

**Footnote:** model further controls for age, age^2^, and sex. Coef* refers to the coefficient in the simplified full model in Appendix 2. Note that ~B4/B5 indcates that these are the corresponding coefficients for the race/ethnicity model (example model is for the SVI).

**Appendix Table 2.2: Interaction coefficients (Ratio of odds ratios) (95% CI) of testing positivity associated with race/ethnicity and neighborhood-level social vulnerability, by wave.**

| **Coef*** | **Race/Ethnicity** | **SVI** | **Wave** | **ROR (95% CI)** |
| --- | --- | --- | --- | --- |
| B6 | . | Middle | Subsequent | 1.69 (1.22-2.35) |
| B7 | . | High | Subsequent | 1.21 (0.86-1.7) |
| B8 | NHB | . | Subsequent | 1.4 (0.81-2.41) |
| B9 | Hispanic | . | Subsequent | 1.18 (0.56-2.48) |
| ~B8/B9 | NHA | . | Subsequent | 0.78 (0.38-1.6) |
| ~B8/B9 | NHO | . | Subsequent | 1.14 (0.44-2.93) |
| ~B8/B9 | Missing | . | Subsequent | 1.21 (0.35-4.22) |
| B10 | NHB | Middle | . | 1.59 (0.91-2.76) |
| B12 | Hispanic | Middle | . | 1.77 (0.84-3.73) |
| ~B10/B12 | NHA | Middle | . | 0.93 (0.4-2.16) |
| ~B10/B12 | NHO | Middle | . | 1.54 (0.55-4.31) |
| ~B10/B12 | Missing | Middle | . | 0.64 (0.17-2.45) |
| B11 | NHB | High | . | 1.14 (0.68-1.89) |
| B13 | Hispanic | High | . | 1.01 (0.51-2.03) |
| ~B11/B13 | NHA | High | . | 0.52 (0.24-1.13) |
| ~B11/B13 | NHO | High | . | 0.39 (0.14-1.06) |
| ~B11/B13 | Missing | High | . | 1.57 (0.53-4.68) |
| B14 | NHB | Middle | Subsequent | 0.67 (0.34-1.31) |
| B16 | Hispanic | Middle | Subsequent | 0.41 (0.16-1.02) |
| ~B14/B16 | NHA | Middle | Subsequent | 0.87 (0.32-2.39) |
| ~B14/B16 | NHO | Middle | Subsequent | 0.67 (0.19-2.41) |
| ~B14/B16 | Missing | Middle | Subsequent | 1.13 (0.2-6.4) |
| B15 | NHB | High | Subsequent | 0.87 (0.47-1.61) |
| B17 | Hispanic | High | Subsequent | 1.01 (0.44-2.34) |
| ~B15/B17 | NHA | High | Subsequent | 1.39 (0.56-3.45) |
| ~B15/B17 | NHO | High | Subsequent | 2.26 (0.67-7.6) |
| ~B15/B17 | Missing | High | Subsequent | 1.47 (0.35-6.13) |

**Footnote:** model further controls for age, age^2^, and sex. Coef* refers to the coefficient in the simplified full model in Appendix 2.

**Appendix Table 2.3: Interaction coefficients (Ratio of odds ratios) (95% CI) for risk of hospitalization**

| **Coef*** | **Race/Ethnicity** | **SVI** | **Wave** | **ROR (95% CI)** |
| --- | --- | --- | --- | --- |
| B6 | . | Middle | Subsequent | 0.82 (0.43-1.55) |
| B7 | . | High | Subsequent | 1 (0.45-2.24) |
| B8 | NHB | . | Subsequent | 0.77 (0.24-2.51) |
| B9 | Hispanic | . | Subsequent | 5.37 (0.52-54.93) |
| ~B8/B9 | NHA | . | Subsequent | 1.66 (0.38-7.27) |
| ~B8/B9 | NHO | . | Subsequent | 0.44 (0.04-4.82) |
| ~B8/B9 | Missing | . | Subsequent | 4.49 (0.32-62.65) |
| B10 | NHB | Middle | . | 0.38 (0.13-1.1) |
| B12 | Hispanic | Middle | . | 0.82 (0.08-8.74) |
| ~B10/B12 | NHA | Middle | . | 1.82 (0.41-8.13) |
| ~B10/B12 | NHO | Middle | . | 0.68 (0.11-4.23) |
| ~B10/B12 | Missing | Middle | . | 1.11 (0.05-25.62) |
| B11 | NHB | High | . | 0.6 (0.23-1.55) |
| B13 | Hispanic | High | . | 1.95 (0.2-18.7) |
| ~B11/B13 | NHA | High | . | 2.65 (0.57-12.39) |
| ~B11/B13 | NHO | High | . | 0.89 (0.12-6.81) |
| ~B11/B13 | Missing | High | . | 2.07 (0.16-26.68) |
| B14 | NHB | Middle | Subsequent | 2.51 (0.54-11.77) |
| B16 | Hispanic | Middle | Subsequent | 0.83 (0.05-12.8) |
| ~B14/B16 | NHA | Middle | Subsequent | 1.04 (0.15-7.51) |
| ~B14/B16 | NHO | Middle | Subsequent | 9.81 (0.47-205.88) |
| ~B14/B16 | Missing | Middle | Subsequent | 0.53 (0.01-23.35) |
| B15 | NHB | High | Subsequent | 1.52 (0.37-6.23) |
| B17 | Hispanic | High | Subsequent | 0.21 (0.01-2.97) |
| ~B15/B17 | NHA | High | Subsequent | 0.71 (0.09-5.35) |
| ~B15/B17 | NHO | High | Subsequent | 2.34 (0.1-54.95) |
| ~B15/B17 | Missing | High | Subsequent | 0.72 (0.03-16.24) |

**Footnote:** model further controls for age, age^2^, and sex. Coef* refers to the coefficient in the simplified full model in Appendix 2.

**Appendix Table 2.4: Interaction coefficients (Ratio of odds ratios) (95% CI) for in-hospital mortality**

| **Coef*** | **Race/Ethnicity** | **SVI** | **Wave** | **ROR (95% CI)** |
| --- | --- | --- | --- | --- |
| B6 | . | Middle | Subsequent | 1.06 (0.93-1.2) |
| B7 | . | High | Subsequent | 1.1 (0.96-1.25) |
| B8 | NHB | . | Subsequent | 0.61 (0.48-0.77) |
| B9 | Hispanic | . | Subsequent | 0.97 (0.71-1.33) |
| ~B8/B9 | NHA | . | Subsequent | 0.86 (0.63-1.18) |
| ~B8/B9 | NHO | . | Subsequent | 0.86 (0.6-1.23) |
| ~B8/B9 | Missing | . | Subsequent | 0.78 (0.57-1.07) |
| B10 | NHB | Middle | . | 1.04 (0.83-1.3) |
| B12 | Hispanic | Middle | . | 1.57 (1.15-2.16) |
| ~B10/B12 | NHA | Middle | . | 1.56 (1.09-2.22) |
| ~B10/B12 | NHO | Middle | . | 1.38 (0.93-2.04) |
| ~B10/B12 | Missing | Middle | . | 0.95 (0.66-1.35) |
| B11 | NHB | High | . | 1.13 (0.92-1.39) |
| B13 | Hispanic | High | . | 1.9 (1.43-2.54) |
| ~B11/B13 | NHA | High | . | 2.19 (1.59-3.01) |
| ~B11/B13 | NHO | High | . | 1.42 (1-2) |
| ~B11/B13 | Missing | High | . | 1.79 (1.32-2.42) |
| B14 | NHB | Middle | Subsequent | 0.94 (0.71-1.26) |
| B16 | Hispanic | Middle | Subsequent | 0.62 (0.42-0.91) |
| ~B14/B16 | NHA | Middle | Subsequent | 0.97 (0.63-1.5) |
| ~B14/B16 | NHO | Middle | Subsequent | 0.69 (0.42-1.13) |
| ~B14/B16 | Missing | Middle | Subsequent | 1.03 (0.67-1.59) |
| B15 | NHB | High | Subsequent | 0.87 (0.67-1.13) |
| B17 | Hispanic | High | Subsequent | 0.42 (0.3-0.6) |
| ~B15/B17 | NHA | High | Subsequent | 1.08 (0.73-1.58) |
| ~B15/B17 | NHO | High | Subsequent | 0.83 (0.54-1.27) |
| ~B15/B17 | Missing | High | Subsequent | 0.53 (0.36-0.78) |

**Footnote:** model further controls for age, age^2^, sex, and hospital. Coef* refers to the coefficient in the simplified full model in Appendix 2.

**Appendix Table 2.5: Results of the joint-significance testing comparing SVI-based disparities between first wave and subsequent waves, within each racial/ethnic group.**

| **Outcome** | **Race/Ethnicity** | **p-value** |
| --- | --- | --- |
| **Testing Positivity** | NHW | 0.373 |
|  | NHB | 0.657 |
|  | Hispanic | <0.001 |
|  | NHA | 0.549 |
|  | NHO | 0.426 |
|  | Missing | <0.001 |
| **Hospitalization** | NHW | 0.007 |
|  | NHB | 0.037 |
|  | Hispanic | 0.007 |
|  | NHA | 0.022 |
|  | NHO | 0.008 |
|  | Missing | 0.030 |
| **In-hospital mortality** | NHW | 0.807 |
|  | NHB | 0.836 |
|  | Hispanic | 0.554 |
|  | NHA | 0.967 |
|  | NHO | 0.654 |
|  | Missing | 0.960 |

**Footnote:** model further controls for age, age^2^, and sex. See Appendix 2 above for details on how p-values are obtained.

**Appendix 3: results of sensitivity analyses**

**Appendix Table 3.1: Odds ratios (95% CI) of in-hospital mortality associated with race/ethnicity and neighborhood-level social vulnerability considered separately, by wave, restricting the analysis to individuals tested in the UPHS system or individuals hospitalized on March 20^th^, 2020, onwards.**

|  |  | **Main Analysis (Table 2)** | | **Restricting to those tested in the UPHS system** | | **Restricted to March 20^th^, 2020, onwards** | |
| --- | --- | --- | --- | --- | --- | --- | --- |
|  | **Category** | **1^st^ Wave** | **2^nd^+ Wave** | **1^st^ Wave** | **2^nd^+ Wave** | **1^st^ Wave** | **2^nd^+ Wave** |
|  | **N** | 2045 | 3749 | 1717 | 3001 | 2026 | 3749 |
| **Race Ethnicity** | **NH White** | 1 (Ref.) | 1 (Ref.) | 1 (Ref.) | 1 (Ref.) | 1 (Ref.) | 1 (Ref.) |
|  | **NH Black** | 0.88 (0.63-1.22) | 1.10 (0.79-1.54) | 0.84 (0.59-1.2) | 1.22 (0.84-1.76) | 0.84 (0.6-1.18) | 1.1 (0.79-1.54) |
|  | **Hispanic** | 0.76 (0.42-1.38) | **1.75 (1.01-3.02)** | 0.8 (0.44-1.47) | 2.1 (1.19-3.7) | 0.75 (0.41-1.36) | 1.75 (1.01-3.02) |
|  | **NH** **Asian** | 1.08 (0.59-1.98) | **1.68 (1.01-2.79)** | 0.82 (0.4-1.66) | 1.85 (1.09-3.17) | 1.06 (0.58-1.94) | 1.68 (1.01-2.79) |
|  | **NH-Other** | 1.21 (0.55-2.66) | 1.52 (0.67-3.45) | 1.21 (0.51-2.85) | 1.28 (0.49-3.34) | 1.21 (0.55-2.68) | 1.52 (0.67-3.45) |
|  | **Missing** | 1.27 (0.49-3.26) | **3.86 (1.82-8.17)** | 1.15 (0.4-3.28) | 4.28 (1.74-10.55) | 1.24 (0.48-3.19) | 3.85 (1.82-8.15) |
|  | **p-val*** | 0.186 | | 0.062 | | 0.161 | |
| **Social Vulnerability** | **Low** | 1 (Ref.) | 1 (Ref.) | 1 (Ref.) | 1 (Ref.) | 1 (Ref.) | 1 (Ref.) |
|  | **Medium** | 0.99 (0.67-1.46) | 1.08 (0.76-1.54) | 0.99 (0.66-1.51) | 1.07 (0.72-1.6) | 0.99 (0.67-1.45) | 1.08 (0.76-1.54) |
|  | **High** | 0.95 (0.65-1.39) | 1.05 (0.73-1.51) | 0.9 (0.6-1.35) | 1.12 (0.75-1.68) | 0.91 (0.62-1.33) | 1.05 (0.73-1.50) |
|  | **p-val*** | 0.903 | | 0.642 | | 0.841 | |

**Footnote:** models control for age, age^2^, sex, and hospital (for in-hospital mortality). Race/ethnicity and SVI results come from different models. Testing positivity refers to the odds of ever testing positive; hospitalization refers to the odds of being hospitalized 2 weeks prior or 4 weeks after the first positive test; in-hospital mortality refers to the odds of dying in-hospital by 30 days after admission with a COVID-19 confirmed flag. *p-val refers to the p-value for interaction between wave and race/ethnicity or SVI.

**Appendix Table 3.2: Odds ratios (95% CI) of intensive care unit stay, and invasive mechanical ventilation associated with race/ethnicity and neighborhood-level social vulnerability, by wave, restricting the analysis to individuals tested in the UPHS system.**

|  |  | **ICU Stay (OR, 95% CI)** | | | | | | |
| --- | --- | --- | --- | --- | --- | --- | --- | --- |
|  |  | **Main Analysis** | | | **Restricting to those tested in the UPHS system** | | **Restricted to March 20^th^, 2020, onwards** | |
|  | **Category** | **1^st^ Wave** | | **2^nd^+ Wave** | **1^st^ Wave** | **2^nd^+ Wave** | **1^st^ Wave** | **2^nd^+ Wave** |
| **Race Ethnicity** | **NH White** | 1 (Ref.) | | 1 (Ref.) | 1 (Ref.) | 1 (Ref.) | 1 (Ref.) | 1 (Ref.) |
|  | **NH Black** | **1.4 (1.1-1.78)** | | 0.94 (0.78-1.12) | 1.45 (1.11-1.9) | 0.91 (0.74-1.11) | **1.4 (1.1-1.79)** | 0.94 (0.79-1.12) |
|  | **Hispanic** | **1.62 (1.13-2.3)** | | **1.53 (1.15-2.04)** | 1.83 (1.25-2.67) | 1.59 (1.15-2.18) | **1.63 (1.14-2.32)** | **1.53 (1.15-2.04)** |
|  | **NH** **Asian** | **1.76 (1.11-2.78)** | | 0.87 (0.61-1.25) | 1.63 (0.97-2.74) | 0.93 (0.63-1.36) | **1.77 (1.12-2.8)** | 0.87 (0.61-1.25) |
|  | **NH Other** | 1.58 (0.87-2.88) | | 0.89 (0.55-1.46) | 1.79 (0.9-3.56) | 0.99 (0.57-1.7) | 1.41 (0.76-2.63) | 0.9 (0.55-1.46) |
|  | **Missing** | **2.35 (1.21-4.56)** | | **3.34 (1.93-5.77)** | 2.45 (1.16-5.19) | 2.36 (1.23-4.54) | **2.37 (1.22-4.59)** | **3.34 (1.93-5.78)** |
|  | **p-val*** | **0.014** | | | 0.055 | | **0.018** | |
| **SVI** | **Low** | 1 (Ref.) | | 1 (Ref.) | 1 (Ref.) | 1 (Ref.) | 1 (Ref.) | 1 (Ref.) |
|  | **Medium** | **1.51 (1.1-2.07)** | | 1.04 (0.84-1.28) | 1.56 (1.09-2.24) | 1.02 (0.79-1.31) | **1.56 (1.13-2.15)** | 1.04 (0.84-1.28) |
|  | **High** | **1.64 (1.23-2.18)** | | 0.86 (0.7-1.06) | 1.91 (1.37-2.66) | 0.9 (0.7-1.15) | **1.69 (1.26-2.25)** | 0.87 (0.7-1.07) |
|  | **p-val*** | **<0.001** | | | **<0.001** | | **<0.001** | |
|  |  | **Mechanical Ventilation (OR, 95% CI)** | | | | | | |
|  |  | **Main Analysis** | | | **Restricting to those tested in the UPHS system** | | **Restricted to March 20^th^, 2020, onwards** | |
|  | **Category** | **1^st^ Wave** | **2^nd^+ Wave** | | **1^st^ Wave** | **2^nd^+ Wave** | **1^st^ Wave** | **2^nd^+ Wave** |
| **Race Ethnicity** | **NH White** | 1 (Ref.) | 1 (Ref.) | | 1 (Ref.) | 1 (Ref.) | 1 (Ref.) | 1 (Ref.) |
|  | **NH Black** | 1.2 (0.88-1.62) | 0.89 (0.68-1.15) | | 1.3 (0.93-1.81) | 0.99 (0.74-1.34) | 1.2 (0.88-1.63) | 0.89 (0.69-1.15) |
|  | **Hispanic** | **1.69 (1.1-2.6)** | **1.85 (1.24-2.74)** | | **1.9 (1.2-3.01)** | **1.98 (1.27-3.1)** | **1.72 (1.12-2.64)** | **1.85 (1.24-2.75)** |
|  | **NH** **Asian** | **2.14 (1.29-3.54)** | 1.24 (0.78-1.95) | | **1.91 (1.06-3.43)** | 1.45 (0.89-2.37) | **2.15 (1.3-3.58)** | 1.24 (0.78-1.96) |
|  | **NH Other** | 1.54 (0.77-3.09) | 1.46 (0.8-2.66) | | 1.44 (0.63-3.3) | 1.64 (0.83-3.25) | 1.45 (0.71-2.96) | 1.47 (0.81-2.67) |
|  | **Missing** | **2.23 (1.08-4.61)** | **4.3 (2.38-7.76)** | | 1.89 (0.8-4.45) | **3.15 (1.47-6.74)** | **2.25 (1.08-4.65)** | **4.3 (2.38-7.77)** |
|  | **p-val*** | 0.138 | | | 0.566 | | 0.142 | |
| **SVI** | **Low** | 1 (Ref.) | 1 (Ref.) | | 1 (Ref.) | 1 (Ref.) | 1 (Ref.) | 1 (Ref.) |
|  | **Medium** | **1.47 (1-2.16)** | 0.98 (0.73-1.33) | | **1.62 (1.05-2.5)** | 1.06 (0.74-1.51) | **1.5 (1.02-2.21)** | 0.99 (0.73-1.34) |
|  | **High** | 1.17 (0.82-1.68) | 0.76 (0.57-1.02) | | 1.42 (0.95-2.14) | 0.9 (0.64-1.27) | 1.19 (0.83-1.72) | 0.77 (0.58-1.03) |
|  | **p-val*** | 0.119 | | | 0.163 | | 0.112 | |

**Footnote:** models control for age, age^2^, sex, and hospital. Race/ethnicity and SVI results come from different models. Outcomes refer to the odds of ICU stay or invasive mechanical ventilation during the first hospitalization with a COVID-19 confirmed flag. . *p-val refers to the p-value for interaction between wave and race/ethnicity or SVI.

**Appendix Figure 1: Flowchart describing study population**
